# The Surgical Treatment of Purulent Pericarditis

**Published:** 1891-07-04

**Authors:** 


### SURGERY.

### THE SURGICAL TREATMENT OF PURULENT PERICARDITIS.

Surgery is everywhere invading domains hitherto sacred to the physician, and regions and organs that once were regarded as for all time beyond the reach of the surgeon's knife are now to be looked upon as coming under treatment by the ordinary principles of surgery. It would almost seem that, the less regard the skilful surgeon pays to the peritoneum, the happier are the results of operations involving that serous sac. And to the various departments of surgery we shall probably, ere long, have to add one of cardiac surgery. Already cases have been recorded where the cavities of the heart have been aspirated successfully, that is, as an operation, although the fact that they have only been resorted to when the patients have been "*in articulo mortis*" naturally precludes any array of successful results. *Paracentesis pericardii* was advocated more than two hundred years ago by Riolanus for cases of purulent pericarditis. Hindenlang, in a contribution on paracentesis pericardii, refers to the first case recorded in which his operation was adopted by Romero in 1819. The results of such half-measures, surgically speaking, have been unsatisfactory when compared with the brilliant results of abdominal surgeons in peritoneal abscesses, and, therefore, the bolder measures adopted by Dr. Bronner, and communicated to the Bradford Med. Chi. Society are a distinct encouragement to apply the ordinary rules of surgery to the pericardium in such cases. E. S., aged 11, a healthy girl, who had up to the time enjoyed perfect health, awoke ill on February 15th, 1890, during the prevalence of the influenza epidemic. Her temperature was 101·5, pulse 100. After several days of very severe illness, the cause of which it was impossible to definitely define, a small area of tubular-breathing was discovered under the angle of the right scapula, but no crepitation. This increased, and the temperature rose to 104° by the ninth day; was reduced by antipyrin, but rose again. The same occurred several times on successive days, the child being in a very bad state. On the morning of the eleventh day there was typical crepitatio redux all over the area of the lower lobe of the right lung. The area was dull on percussion, and while crepitation was diminishing, the dulness gradually became more marked, and it became evident that there was pus forming in the right pleura. This was confirmed by aspiration on March 4th, the sixteenth day of the illness, and on March 7th an incision was made in the sixth intercostal space, behind the axillary line. A pint and a-half of sweet pus was withdrawn, and two drainage tubes inserted side by side for the purpose of eventual irrigation. As three days after the operation the temperature did not go down, the cavity was washed out daily with 5 per cent. boric acid solution, until the fluid ran off clear. Still pulse and respirations did not diminish, being 140 and 45 respectively. There was clearly some further mischief which Dr. Bronner and Dr. Major, who had operated, had not discovered. On March 18th Dr. Major noticed a slight increase in the area of cardiac dulness over the heart and faintness of the heart sounds, and mooted the possibility of fluid in the pericardium. In the course of the following two days dyspnœa increased very markedly, and the increase of the area of cardiac dulness over the pericardium became unmistakable. On the morning of March 20th the child woke up with severe dyspnœa, lasting a quarter of an hour. The pulse was scarcely to be felt. Dr. Major advised speedy aspiration of the pericardial fluid and incision, with subsequent drainage in case the fluid should be pus, which in all probability it would be. In the afternoon, Mr. Pridgin Teale saw the child, with Dr. Bronner and Dr. Major, and confirmed the presence of fluid in the pericardium. After the opening into the right pleura had been closed as a matter of precaution, the child was chloroformed, the pulse going down from 160 to 60, and becoming full and bounding. A trochar was passed through the fourth intercostal space, one inch to the left of the sternum, in order to avoid the internal mammary artery. Pus appeared. Mr. Teale then made an incision outwards, using the trochar as a director, and carefully watching for any indication of the heart touching the point of the knife. Nearly two pints of sweet thick pus slowly escaped through the opening. The apex of the heart could afterwards be felt beating against the tip of the little finger inserted into the opening. A short drainage tube was left in the wound and sublimate gauze pads used as a dressing. At no time during the operation were any ill effects of any kind noticed in the heart's action. The relief afforded by the operation was very great. The child, which before had been sitting bolt upright, could now lie flat on its back, and slept in this position. The temperature, however, which for over eight days had been below 100 deg., rose to 102 deg. in the evening. The pulse and respiration did not diminish, although there had been much discharge from the wound. The tube slipped out and was replaced and held in place by a strip of gauze. On March 22, as the temperature, pulse, and respiration had not diminished, grs. x. iodoform, suspended in previously sterilised glycerine and water (Billroth's formula ℞. iodoform grs. 50, glycerine m. 80, pulv. acaceæ grs. viii, aq.dist. ad ℥i) were injected into the pericardial cavity through the drainage tube. The injection and the process of dressing caused no discomfort or ill-effect whatever, and did not influence the pulse in the least.

As no improvement took place it was decided on March 31st, the eleventh day after the operation, to syringe out the pericardial cavity, although the pus had always been sweet, a step which, without previous experience, was adopted with some diffidence, as the pulse had become very weak, and was at times slightly irregular. Two tubes were inserted side by side to give free egress to the fluid and to prevent any pressure on the heart, and a 5 per cent. boric acid solution of 100° was run through with a douche at a minimum pressure till it ran out clear through the free tube in jets synchronous with the heart beat. This had no ill effect on the pulse, nor did the daily repetition ever seem to have any ill effect whatever. The child used to sit up during the dressing, lending a helping hand and chatting and joking. Once, when a small proportion (1 in 500) of carbolic acid was added to the boric acid solution, the patient complained of faintness and queerness. The pulse and respirations and temperature, however, did not improve, and in spite of every care and of the administration of suitable remedies the child sank and died on April 15th. Dr. Bronner's case was undoubtedly handicapped by the numerous other complications, but though the result was not in recovery, it is a most instructive case, and one which gives every encouragement to repeat the operation in suitable cases, and therefore we need offer no apology for quoting him *in extenso*. He further refers to the admission of air into the pericardial sac, and the irrigation with boric acid, which never seemed to affect the heart's action in the least, and the case without doubt goes to prove that the dangers of surgical interference with the pericardium are no counter-indication where the interference is called for; in this respect corroborating the two successful cases of Rosenstein and West. The former incised and drained the pericardium of a boy aged 10. The wound was closed on the nineteenth day, and perfect recovery took place. West was successful in the case of a boy age 16, the patient recovering perfectly in five weeks.

We firmly believe that Dr. Bronner and those who assisted him prolonged the life of the patient by the bold and skilfully-conducted operation we have related above, and which was resorted to only after the most careful consideration, and when otherwise all hope of recovery had vanished.

---

DR. WYETH, of New York, pays a high tribute of praise to the method of administering ether advocated some time ago by Mr. Teale. He states that he had been giving ether now for twenty years or more, and never knew how to administer it properly until within a year, when his attention was drawn to the apparatus recommended by Mr. Teale. He at once secured it, and found that its use as an inhaler removed all possibility of danger from an excessive amount of the anæsthetic.

